# Statistical Classification for Raman Spectra of Tumoral Genomic DNA

**DOI:** 10.3390/mi13091388

**Published:** 2022-08-25

**Authors:** Claudio Durastanti, Emilio N. M. Cirillo, Ilaria De Benedictis, Mario Ledda, Antonio Sciortino, Antonella Lisi, Annalisa Convertino, Valentina Mussi

**Affiliations:** 1 Dipartimento di Scienze di Base e Applicate per l’Ingegneria, Sapienza Università di Roma, Via A. Scarpa 16, 00161 Roma, Italy; 2 Institute of Translational Pharmacology, CNR, Via del Fosso del Cavaliere, 00133 Roma, Italy; 3 Institute for Microelectronics and Microsystems, CNR, Via del Fosso del Cavaliere, 00133 Roma, Italy

**Keywords:** tumoral genomic DNA, Raman spectroscopy, classification, principal component analysis, logistic regression, minimum distance classifiers

## Abstract

We exploit Surface-Enhanced Raman Scattering (SERS) to investigate aqueous droplets of genomic DNA deposited onto silver-coated silicon nanowires, and we show that it is possible to efficiently discriminate between spectra of tumoral and healthy cells. To assess the robustness of the proposed technique, we develop two different statistical approaches, one based on the Principal Components Analysis of spectral data and one based on the computation of the $\ell^{2}$ distance between spectra. Both methods prove to be highly efficient, and we test their accuracy via the Cohen’s κ statistics. We show that the synergistic combination of the SERS spectroscopy and the statistical analysis methods leads to efficient and fast cancer diagnostic applications allowing rapid and unexpansive discrimination between healthy and tumoral genomic DNA alternative to the more complex and expensive DNA sequencing.

## 1. Introduction

Today, it is widely accepted that cancers result from changes in the nucleotide sequence due to unrepaired DNA damage [1]. Identifying circulating tumor DNA in human body fluids, blood in primis can indeed favor the development of promising approaches for early disease diagnosis and personalized therapies [2,3]. Currently, the most popular molecular genetic technology is based on DNA sequencing methods which require expensive and complex enzyme based target or signal amplification procedures, as well as the risk of false positive or false negative identifications, still prevent genetic analysis to be introduced in the routine clinical. Therefore, there is an urgent need of innovative, low-cost, easy and fast approaches which allow for identifying the DNA changes.

The relevance of Raman spectroscopy in medical diagnostics [4], and in particular in the study of cancer diseases [5], has been widely pointed out in the recent pertinent literature. The potentiality of this technique lies in its label–free character, which allows for directly analyzing biological samples and obtaining unaltered information about their physico–chemical properties.

Here, we exploited Raman spectroscopy to investigate aqueous droplets of genomic DNA deposited onto silver-coated silicon nanowires (Ag/SiNWs). By following the same experimental procedure proposed in [6], whose proposal consists of the use of a platform based on silicon nanowires (SiNWs) to interrogate DNA, we use Raman mapping to collect several spectra that are statistically analyzed here to discriminate between samples extracted from tumoral and healthy cells.

Raman spectroscopy is an inelastic optical scattering technique, which records the light scattered from vibrations in molecules or optical phonons in solids [7,8]. These inelastic scattering processes have a small cross section and, thus, the typical intensity of Raman signals is very low, as discussed for example in [9]. On the other hand, as it was firstly shown in [10], due to electromagnetic and chemical effects, the Raman signal coming from molecules adsorbed on a metal nanostructure can be increased by several orders of magnitude. This phenomenon, known as Surface-Enhanced Raman Scattering (SERS), as shown for instance in [11,12], has been exploited in our experiment by dripping the DNA aqueous solution on a substrate made of a disordered array of silver-coated silicon nanowires, whose effectiveness in enhancing the Raman signal has been recently demonstrated in a series of experimental studies; we refer, for example, to [13,14,15,16,17,18,19] and the references therein.

In this way, we are able to collect Raman maps of the deposited drops composed of several good quality Raman spectra, which, although very similar among each other, allow us to distinguish between drops of healthy and tumoral DNA. Anyway, the major challenge of this approach is that the Raman mapping generates large data sets that need an advanced data processing to extract meaningful information allowing us the discrimination between the healthy and tumor DNA sample.

The aim of this work is thus to build a binary classification model aimed to discriminate, with high accuracy, spectra coming from different DNA molecules, corresponding to the outcomes of a target variable taking values 0 and 1 for healthy and tumoral samples, respectively.

Our proposal is based on two different methods. The first one reduces initially the amount of predictors by means of a Principal Components Analysis (PCA). After reducing dimensionality, the new variables are exploited to build a logistic regression model, whose outcome is the desired classifier. The second method involves the full data set to exploit the geometric features of the Raman spectra. Indeed, the classifier adopted in this case is based on the computation of the $\ell^{2}$ distance between test samples and the spectral average of healthy and tumoral training spectra. We will show that both of the strategies achieve a very high accuracy, close to 90%.

The paper is organized as follows: in Section 2, we discuss experimental and statistical methods, mainly focusing on the latter. Our results are discussed in Section 3, whereas, in Section 4, we summarize our conclusions.

## 2. Methods

In this section, we describe the processes and approaches that we have developed in the different steps of our study. We shall provide a very short account of the sample preparation procedure and the Raman measurements, referring to [6] for a thorough and detailed description. On the other hand, we shall discuss in detail the statistical approach developed to analyze the experimental data, which is the true novel contribution provided in this paper.

### 2.1. Experimental Procedures

Plasma enhanced chemical vapor deposition (PECVD) was used to grow Au catalyzed SiNWs on Si wafers, kept at 350 °C, using SiH${}_{4}$ and H${}_{2}$ as precursors. The coating was realized by evaporating an Ag film onto SiNWs arrays with a nominal thickness of 100 nm.

Following the skin cancer model given by [20], we used the human melanoma cell line SK-MEL-28, which was compared to the human immortalized keratinocyte HaCaT as a control health skin model. After standard culture, harvesting, and centrifugation, cell pellets were obtained and used to extract the genomic DNA that, in turn, was re-suspended in DNase free water to obtain a 20 ng/μL solution.

The final samples are prepared by depositing one drop of the DNA solution on Ag/SiNWs substrates coming from the very same batch.

Each droplet is spectrally mapped after drying by means of a DXR2xi Thermo Fisher Scientific Raman Imaging Microscope equipped with a 532 nm excitation laser, to better exploit the plasmon resonance of nanostructured silver, and a 50× objective. For each droplet, Raman spectra are collected at points on a square grid with spacing 4μm, at 1 mW laser power and performing four accumulations lasting 5 ms each, for a total of about 2000 spectra per droplet. The entire comparison experiment has been repeated 10 times, by analyzing 10 droplets of healthy DNA, and ten of cancer DNA. For each comparison experiment, the two measured droplets came from a new pellet of corresponding cells.

As far as the cell culture is concerned, the human melanoma cell line SK-MEL-28, routinely used in skin cancer research and able to form tumors in nude mice, was used as a skin cancer model and compared with the human immortalized keratinocyte cell line, HaCaT (American Type Culture Collection, ATCC), which was chosen as the control health skin model [21,22]. The cell lines were cultured in complete Dulbecco’s modified Eagle’s medium (DMEM; Hyclone, South Logan, UT, USA) with high glucose (4.5 g/L), supplemented with 10% fetal bovine serum (FBS, HyClone), 2 mM-glutamine, and 100 IU/mL penicillin/streptomycin (Invitrogen, Carlsbad, CA, USA). The cells were kept in a humidified atmosphere with 5% CO${}_{2}$ at 37 ${}^{\circ }$C, passaged every 3–4 days at a sub-cultivation ratio of 1:5 and used within 5–20 passages. Regarding the DNA isolation, SK-MEL-28 and HaCaT cells, after medium removal, were harvested by trypsin treatment and an amount of 2–3 × 10^6^ were centrifuged for 5 min at 4000 rpm. The resulting cell line pellets were processed to extract the genomic DNA. The cells were lysed in 1 mL of hypotonic lysis buffer (HEPES 10 mM, MgCl 1.5 mM, KCl 10 mM, and fresh Ditiotreitolo 5 mM), incubated 15 min in ice, and centrifuged for 10 min, at 2000 rpm and 4 ${}^{\circ }$C. To extract the genomic DNA, the pellet samples were incubated for 1 h at 37 ${}^{\circ }$C in 750 μL of nuclear lysis buffer (Tris-HCl 10 mM, NaCl 400 mM, EDTA 2 mM, 75 μL SDS 10%, 25 μL of 10 μg/μL proteinase-K), treated with 250 μL of NaCl 6 M and centrifuged for 15 min at 2000 rpm and 4 ${}^{\circ }$C. The supernatants containing genomic DNA were recovered and then precipitated adding a double volume of EtOH 100% and centrifuged for 10 min at 2000 rpm and 4 ${}^{\circ }$C. The DNA pellets were washed in EtOH 70%, centrifuged for 10 min at 7500 rpm and 4 ${}^{\circ }$C and re-suspended in 100–200 μL of DNase free H${}_{2}$O. The DNA concentrations were measured with a spectrophotometer (Eppendorf BioSpectrometer® basic) by reading absorbance at 260 nm, and 260/280 ratio absorbance was checked to assess the purity of the DNA. The DNA concentration was kept constant throughout the entire study at ca. 20 ng/μL. This concentration was selected in order to achieve sufficient signal-to-noise ratio for both Raman spectra and fluorescence image after staining with Hoechst 33,342 solution. To exclude any influence of the unavoidable morphological variations related to different fabrication batches of the Ag/SiNWs, for each experiment, we deposited two drops, containing respectively HaCaT and SK-MEL-28 cell DNA, on fresh substrates from the very same batch. In addition, we repeated each experiment for three times by using different DNA samples from SK-MEL-28 and HaCaT cells.

### 2.2. Data Set and Conveyed Information

**Figure 1:**
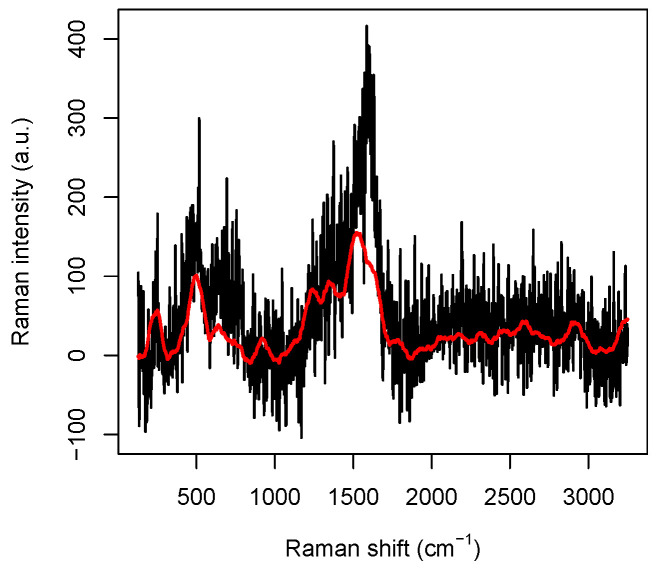
Comparison between a raw Raman spectrum and the curve (in red) obtained by performing Savitzky–Golay filtering.

As a data set, we have considered *n* = 3980 spectra, 1990 from the tumoral and 1990 from the healthy DNA droplet. The spectra have been randomly chosen in the central part of the droplets, since in this region the biological layer formed after dehydration happens to be thinner, so that the interaction between the single molecules and the nanostructured substrate is direct and tighter, and the SERS effect more effective, ensuring a good quality of the Raman spectra. Due to the fact that the spectra are collected at points of the droplet at distance larger than, or equal to, 4 μm while the size of a DNA molecule is of a nanometer order, we can reasonably assume that our data are independent. A typical Raman spectrum we will deal with is reported in Figure 1. In order to explain its main features, we have to review some basic facts about Raman spectroscopy. In Raman spectroscopy, incident photons are scattered by molecules in such a way that the emitted photon has energy different from that of the incident one and molecules, after the interaction, jump to a different vibrational energetic level. This phenomenon is often explained saying that the molecule absorbs the incident photon and jumps to a “virtual” energy level with an incredibly short life-time (say less than 10^−15^ s); then, it relaxes to a vibrational energy state different from the initial one emitting a photon, which, consequently, has energy different from that of the incident one. The total emitted energy, suitably normalized and expressed in arbitrary units, is called *Raman intensity*, whereas the energy difference between the incident (laser) light and the scattered (detected) light, expressed in cm${}^{-1}$, is called *Raman shift*.

Raman spectroscopy is a powerful method in investigating biological systems because it provides a molecular fingerprint of the samples in a completely label-free way and with high specificity [4]. Indeed, the peaks appearing in the spectra are uniquely associated with particular chemical structure present in the molecule. For instance, in the raw spectrum reported in Figure 1, where no pre-processing has been performed on the data obtained from the spectrometer, some peaks are perfectly visible and can be roughly located at 230 cm${}^{-1}$, 540 cm${}^{-1}$, 1320 cm${}^{-1}$, 1570 cm${}^{-1}$, and 2930 cm${}^{-1}$. Very precise measures of the peak positions can be performed by suitably averaging several spectra, as shown in [6], Supplementary Materials, Figure S5, but we note that, even in the data reported in Figure 1, the CH-group vibration peak at 2930 cm${}^{-1}$ and the Ag-N stretching vibration mode at 230 cm${}^{-1}$ are perfectly visible.

As regards the diagnostic potential of Raman spectroscopy, cancer is nowadays associated with changes in the nucleotide sequence of DNA molecules [1] which also induces variations in DNA physical properties, such as stiffness, length, and shape. It has been demonstrated in [6], in the same experimental set–up considered herethat the variation of these physical properties causes modifications in the interaction between DNA molecules and the nanostructured substrate which is reflected in the observed Raman spectra.

### 2.3. Data Pre-Processing

Each Raman spectrum here considered consists of *p* = 1680 Raman intensity values corresponding to *p*-values of the Raman shift, lying in the interval between 50.6 and 3288.5 cm^−1^. Moreover, we obtained smoothed data by filtering the original raw spectra with the Savitzky–Golay algorithm [23] with a polynomial order 5 (see also [24]) over a window of 90 data points treated as convolution coefficients. In Figure 1, we plotted a raw spectrum and its smoothed version. The original data are kept for the highest and lowest wavenumbers, otherwise truncated by the preprocessing procedure, to avoid losing information at the sides.

Although the large part of the collected spectra share a similar behavior, there are some that are suspiciously different from the others. These spectra have been considered as outliers associated with local experimental fluctuations and thus eliminated from the analysis.

We build a decision surface to identify and remove outliers from both the healthy and tumoral data sets by adding and subtracting three times the (point-wise) empirical standard deviations to the average spectra. All the spectral patterns featuring at least a point out of the decision surface are then discarded.

**Figure 2:**
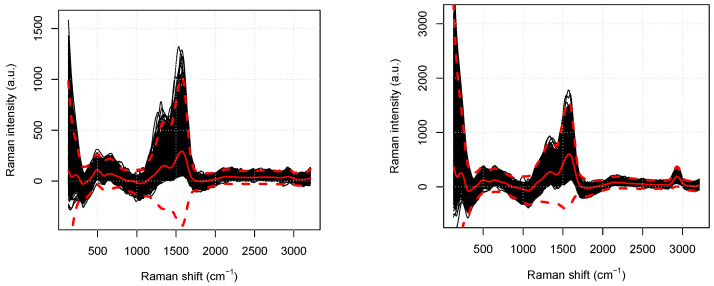
For the healthy (**left**) and tumoral (**right**) data set, we report the smoothed spectra (black solid lines), the average spectra (red solid lines), and the extreme curves computed by adding to and subtracting from the average Raman intensity three times the standard deviation (red dashed lines) labeling the decision surfaces.

Figure 2 shows the whole set of spectra for the healthy and tumoral cell, in the left and in the right panel, respectively. Both the panels show the corresponding decision surfaces, labeled by average spectra (solid lines) and the extreme curves (dashed lines). In both of the cases, the selection procedure allows us to discard approximately 15% of the available spectra.

### 2.4. Two Statistical Approaches

We will propose two different models, one based on the PCA analysis and one based on the computation of $\ell^{2}$ distance. The spectra are split into test and training sets, the first used to tune simultaneously the parameters of both the models proposed here, the second to validate them. As aforementioned, we deal with a binary target variable *W*, whose outcomes 1 and 0 correspond to tumoral and healthy DNA molecules, respectively.

#### The Local Method: PCA Analysis and Logistic Regression

Borrowing the notation from ([25], Chapter 1 and Paragraph 8.2.1), any single spectrum is represented as a column vector $x\in R^{p}$. By collecting the *N* row vectors $x^{†}$, where † denotes transpositions, we construct the N×p matrix X which represents the entire data set. The *j*-th column of X is the collection of the *N* observations of the *j*-th variable, namely, the intensity corresponding to the *j*-th value of the Raman shift.

Thus, we produce the N×p matrix Y by centering X with respect to the columns (i.e., the Raman shift). In more detail, each entry $y_{ij}$ of Y is computed by subtracting from each entry $x_{ij}$ of X the sample mean computed along the elements corresponding to the same Raman shift, that is to say by setting

(1) $$y_{ij}=x_{ij}-\frac{1}{N}\sum_{s=1}^{N}x_{sj}$$

for any i=1,⋯,N and j=1,⋯,p. A *principal components analysis* is then obtained by the eigendecomposition of the empirical covariance matrix $Y^{†}Y$ (see, for example, [26]). The so-called *principal components directions* $v_{1},\cdots ,v_{p}\in R^{p}$ are computed and, as usual, we call *i*-th *principal component loadings* the *p* elements of the column vector $v_{i}$. The projection $z_{i}=Yv_{i}\in R^{N}$ is called the *i*-th *principal component* of the data Y. The variance of each principal component (PC) is given by the corresponding eigenvalue and it concentrates on the first *m* principal components, allowing us to neglect in the next step all the other p−m components.

The selected first *m* principal components $z_{1},\cdots ,z_{m}$ are thus interpolated to build a logistic regression model to estimate the probability mass function of the binary target variable *W* by

(2) $$\mathrm{Pr}\left(W=1|z_{1},\cdots ,z_{m}\right)=\frac{e^{\beta_{0}+\sum_{i=1}^{m}\beta_{i}z_{i}}}{1+e^{\beta_{0}+\sum_{i=1}^{m}\beta_{i}z_{i}}}\;\;\mathrm{and}\;\;\mathrm{Pr}\left(W=0|z_{1},\cdots ,z_{m}\right)=\frac{1}{1+e^{\beta_{0}+\sum_{i=1}^{m}\beta_{i}z_{i}}},$$

where $\beta_{i}\in R$ for i=0,⋯,m. In this case, we consider an optimization parameter λ∈[0,1] such that we associate the outcome for the binary variable W=1 to each set of components $z_{1},\cdots ,z_{m}$ if $\mathrm{Pr}(W=1|z_{1},\cdots ,z_{m})\geq \lambda$ and W=0 otherwise. This method is referred to as “local” since the PCA analysis selects a subset of spectral indices to be the most relevant within the subsequent regression.

### 2.5. The Global Method: Geometric Analysis

The second proposed strategy aims to distinguish spectra coming from the healthy and tumoral data set following their geometric features.

**Figure 3:**
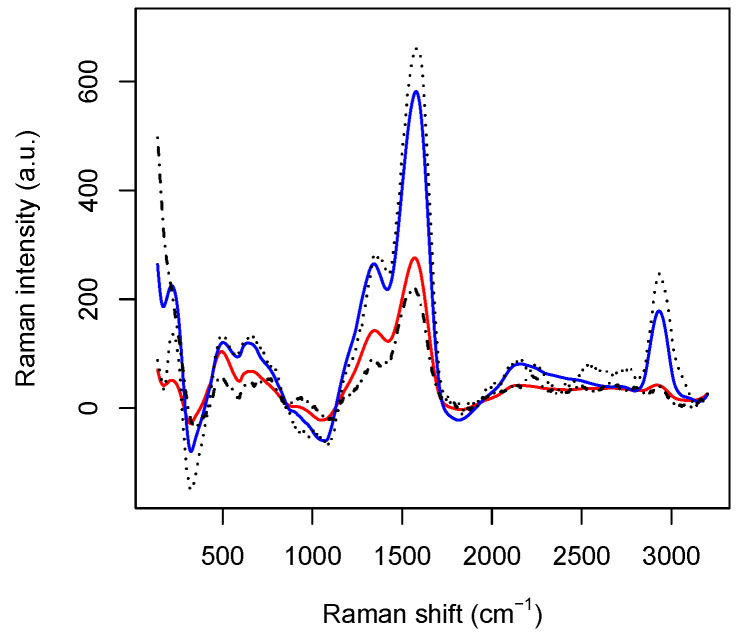
The red and the blue solid lines are, respectively, the average spectrum of the healthy and the tumoral data set. The point-dashed and the dashed lines report, respectively, one healthy and tumoral pre–processed spectrum. For the healthy spectrum, d_*t*_ = 14.9 × 10^6^ and d_*h*_ = 4.7 × 10^6^. For the tumoral spectrum, d_*t*_ = 2.8 × 10^6^ and d_*h*_ = 16.6 × 10^6^.

After splitting the dataset into training and test sets, the training set is used to produce the healthy and the tumoral average spectra, represented by the column vectors *h* and *t* of $R^{p}$ (see also Figure 3). Thus, for each spectrum belonging to the test set represented by the *i*-th row of the data matrix X, we compute the $\ell^{2}$ distances

(3) $$d_{h}\left(i\right)=\sum_{s=1}^{p}|x_{is}-h_{s}{|}^{2}\;\;\mathrm{and}\;\;d_{t}\left(i\right)=\sum_{s=1}^{p}{\left|x_{is}-t_{s}\right|}^{2}.$$

so that each spectrum can be classified by setting the following outcome binary function

(4) $$g_{\mathrm{out}}\left(i\right)=I\left\{\tau d_{t}\left(i\right)\leq \left(1-\tau \right)d_{h}\left(i\right)\right\},$$

where τ∈[0,1] is an optimization parameter. As above, the outcome is equal to 0 if the spectrum is identified as coming from healthy DNA molecules and equal to 1 otherwise. This method is referred as “global” since the $\ell^{2}$ distance is computed over the complete set of spectral data.

## 3. Results and Discussion

The first step of our analysis is concerned with the estimation of the two optimal tuning parameters λ and τ by means of a 10-fold cross-validation procedure, a popular method used to evaluate predictive models over a limited amount of sampled data (see ([27], Ch. 7)). As aforementioned, the tuning parameter λ sets a threshold for the probability mass function of the binary target variable built by means of the logistic regression. Given an input, if the corresponding estimated probability is higher than λ, the sample element is labeled as tumoral, otherwise as healthy. On the other hand, as shown in Equation (4), the tuning parameter τ sets a multiplicative factor to scale the distances between the sample element and the healthy and tumoral means in order to label it.

The original sample is randomly partitioned into 10 equally sized subsamples. A subsample is kept as test set, while the other nine are used as training data. Then, the accuracy of both methods are evaluated, while the cross-validation process is repeated 10 times, paying attention to using each round a different subsample as a test group. The 10 results are then averaged to compute a single estimation. The advantage of this validation strategy is that all observations are used at the same time for both training and testing and each observation is used for testing exactly once.

The accuracy of the choice of the tuning parameters is evaluated by means of the so-called Youden’s *J* statistic, or-simpler, Youden’s index, given by the formula

**Figure 4:**
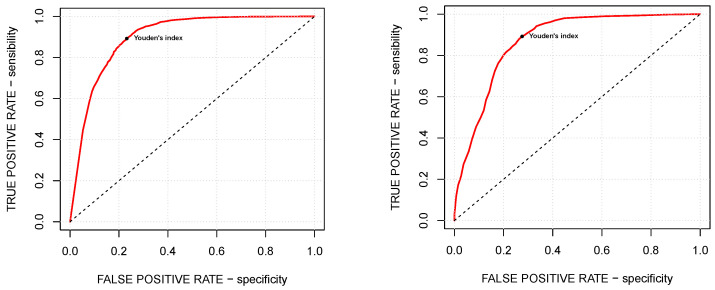
ROC curves for the local (**left panel**) and the global (**right panel**) methods. The markers make evident the corresponding Youden’s indices.

(5) Youden’sindex=sensitivity+specificity−1,

where sensitivity and specificity are the true and false positive rates, respectively. Figure 4 explores the trade-off between specificity and sensitivity by means of the corresponding ROC curve, while Table 1 presents for both of the models the choice of the optimal tuning parameters, corresponding to the maximal Youden’s indices, and the Area Under the Curve (AUC) value, measuring the two-dimensional area under the ROC curve and thus providing an aggregate measure of the performance across all possible choices of the tuning parameters (see, for example, [28]).

As shown in Table 1, both λ and τ are smaller than 1/2, which is the exact balance of the healthy and tumoral subsets. However, it matches our goals since these values of the tuning parameters reduce the amount of false negatives (i.e., not detecting tumoral samples) paying the price of a larger amount of false positives (i.e., misclassifying healthy samples), which seems to be the fairest choice.

**Figure 5:**
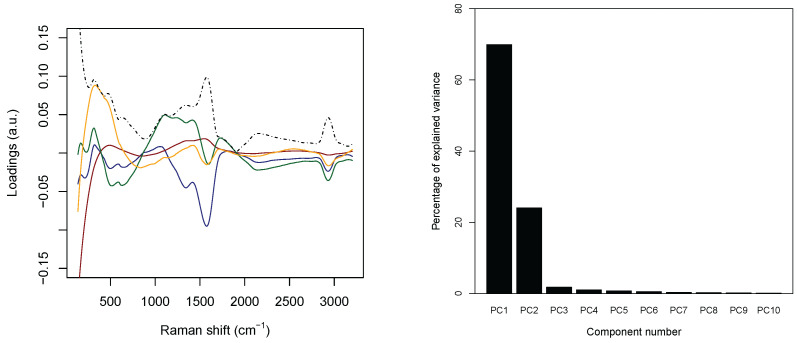
(**Left**) the four solid lines report the loadings associated with the first four principal components (blue first, brown second, green third, and yellow fourth); the dashed black lines are the intensity, i.e., the square root of the sum of the squares, of the loadings of the first four components. (**Right**) percentage of variance as a function of the principal component index.

**Figure 6:**
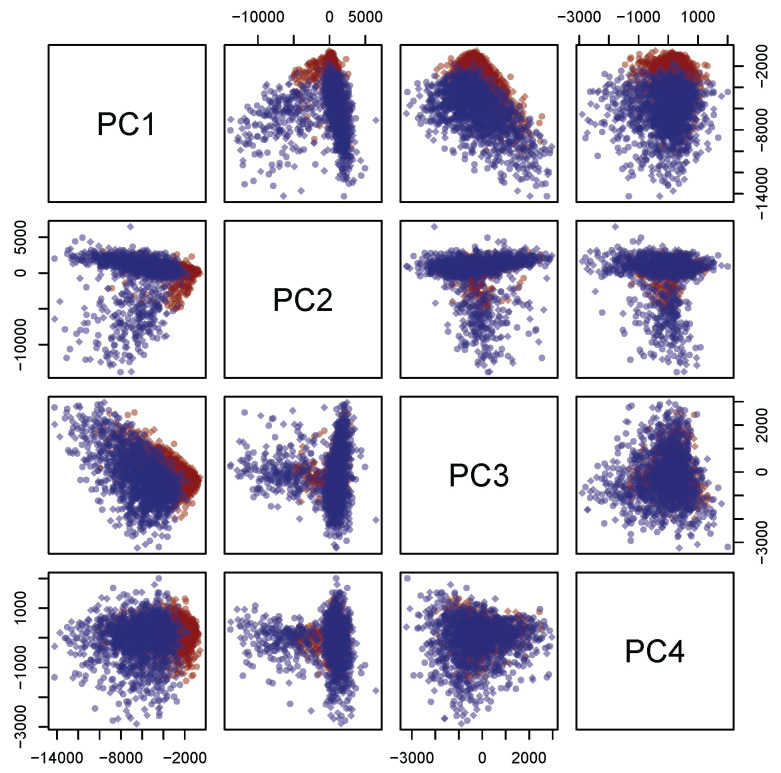
Projections on the coordinate planes of the distribution of the first four principal components on the coordinate planes. The healthy and the tumoral samples are labeled by blue and red points, respectively.

Concerning the PCA analysis, as shown in Table 2 (see the right panel in Figure 5), 97.0% circa of the variance concentrates on the first *m* = 4 principal components. Furthermore, Figure 6 shows a strong separation between the third and fourth principal component. Thus, from now on, only the first four principal components are selected. In addition, each discarded component is characterized by a proportion of variance sensibly smaller than 1% (see again Table 2).

The left panel in Figure 5 collects the loadings, that is, the coefficients of the linear combinations of the original variables defining the principal components, related to the first four PC’s, and, thus provide a measure for the contribution of each observable to the main components. The solid lines are concerned with the first four PC’s separately, while the dashed black line represents the associated intensity, that is, the $\ell^{2}$–norm associated with the loadings of the considered PC’s. The three main peaks appearing in the panel correspond to wavenumber 230 cm${}^{-1}$, 1550 cm${}^{-1}$, and 2930 cm${}^{-1}$. The first one is related to the Ag–N stretching vibration mode. The second one is contributed both by the overlapping of C–C and C–N stretching vibrations involving the aromatic rings of the DNA bases, and the so–called “cathedral peaks”, deriving from the formation of a carboneous layer due to the photo-decomposition of the DNA at the silver surface upon laser irradiation. The last one is linked to the vibrations of the CH-group. While the first and the third peaks can be associated with the fourth and the third principal components, respectively, the first two principal components are related mostly to the second peak, which behaves very differently for healthy and tumoral data (see also Figure 3).

The estimates for the coefficients $\beta_{i}$, i=0,…,4 obtained by the logistic regression are collected in Table 3. Recall that, in this case, the optimization parameter λ∈[0,1] is defined such that W=1 for each $z_{1},\cdots ,z_{4}$ if $\mathrm{Pr}(W=1|z_{1},\cdots ,z_{4})\geq \lambda$, otherwise W=0.

We evaluate, now, the accuracy of the proposed methods by means of the two confusion matrices in Table 4 (see, again, [28]) computed by averaging true positives (TP), true negatives (TN), false positives (FP), and false negatives (FN) over all the possible choices of the training and the test set of data. We use the values collected in Table 4 to calculate, for both methods, the so-called Cohen’s κ statistic, which takes values in [−1,+1] and measures the agreement between two classifiers and can also be used to assess the performance of a classification model, given by the formula (in the binary case)

(6) $$\kappa =\frac{2\left(\mathrm{TP}\times \mathrm{TN}-\mathrm{FP}\times \mathrm{FN}\right)}{\left(\mathrm{TP}+\mathrm{FP}\right)\left(\mathrm{FP}+\mathrm{TN}\right)+\left(\mathrm{TP}+\mathrm{FN}\right)\left(\mathrm{FN}+\mathrm{TN}\right)},$$

see, for example, [29]. For the first method, we obtain $\kappa_{\mathrm{local}}=0.66$, while for the second $\kappa_{\mathrm{global}}=0.62$. Both values make evident a good agreement and accuracy in predictions for both methods.

Now, we aim to evaluate the performance of the combination of the two methods, with the purpose of improving predictions and, at the same time, reducing the variance in the predictions and, thus, enhancing their stability. Table 5 compares the reliability of the predictions of the two models, averaging over the outcomes of the 10-fold cross-validation performed for the optimal tuning parameters. It is important to remark that when the outcomes for both models are either 0 (TN or FN) or 1 (TP or FP), the amount of correct prediction is 92.2% (negative predictive value) and 81% (positive predictive value), respectively. Furthermore, the total proportion of wrong negative outcomes is 2.9%.

Table 6 investigates the accuracy of the predictions. Even if 11% of the predictions are at odds, and thus they should be directly marked as unreliable, 86% (joint accuracy) predictions are valid when both agree.

## 4. Conclusions

Raman spectra of tumoral and healthy genomic DNA have been collected by analyzing aqueous DNA droplets deposited onto silver-coated silicon nanowires. We used, respectively, the human melanoma cell line SK-MEL-28 and the human immortalized keratinocyte HaCaT as tumoral and healthy samples.

Pre-processed spectra were analyzed by means of two different techniques: a PCA based algorithm powered with linear regression and a pure geometric algorithm were devised to predict the tumoral or healthy origin of each spectra. Both algorithms achieve very high accuracy, close to 90%. We also checked accuracy by means of the Cohen’s κ statistic and for both algorithms we found very good values of the Cohen’s κ index—larger than 0.6. As far as the evaluation of the pre-processing method is concerned, we fix the optimal tuning parameters, and then we compare sensitivity and specificity of the pre-processed spectra with the ones characterizing raw data. Regarding the local method, we obtain 0.80 vs. 0.79 and 0.86 vs. 0.81 for specificity and sensitivity, respectively, while we have 0.76 vs. 0.69 (specificity) and 0.84 vs. 0.85 (sensitivity), as far as the global method is considered. We achieve thus a slight improvement for the local method, while we obtain a substantial enhancement for the global method.

On one hand, the PCA approach, rather standard in analyzing Raman measurements, aims at reducing the data set through the analysis of the covariance matrix. On the other hand, the geometric method that we implemented in this paper looks at the whole set of data and is based on the simple computation of the $\ell^{2}$ distance between spectra. The fact that both methods work nicely in discriminating healthy and tumoral molecules is a strong sign of the robustness of the indications provided by the Raman measurements.

In conclusion, we developed a detailed statistical analysis which proves that SERS measurements can be successfully used for efficient and fast cancer diagnostic applications. In particular, our innovative classification approach allows a rapid and unexpansive discrimination between healthy and tumoral genomic DNA, which is based on the different conformation assumed by healthy and cancer DNA upon dehydration on the 3D nanostructured substrate, and thusrepresents a powerful alternative to the more complex and expensive DNA sequencing. The undemanding fabrication technology of the Ag/SiNWs, combined with the potential of Raman analysis and proper data processing methods, makes the proposed approach suitable for cancer diagnostic applications, alternative or complementary to the more complex and expensive DNA sequencing analysis.

## Figures and Tables

**Table 1:** Optimal tuning parameters and AUC for both the methods.

Method	Optimal Tuning Parameter	Area under the Curve
local	λ=0.46	0.899
global	τ=0.34	0.871

**Table 2:** Variance analysis (first eight principal components).

Principal Component	Standard Deviation	Proportion of Variance	Cumulative Proportion
PC1	5157.11860	0.78068	0.78068
PC2	2311.49810	0.15684	0.93752
PC3	866.34106	0.02203	0.95955
PC4	581.91444	0.00994	0.96949
PC5	496.66847	0.00724	0.97673
PC6	405.79667	0.00483	0.98156
PC7	327.32049	0.00314	0.98471
PC8	284.63234	0.00238	0.98708

**Table 3:** Estimates of $\beta_{i}$ and corresponding errors.

*i*	Estimate	Standard Deviation
0	−6.6094526	0.2424116
1	−0.0014903	0.0000540
2	−0.0002585	0.0000440
3	−0.0020081	0.0001042
4	−0.0007470	0.0001154

**Table 4:** Confusion matrix (local method/global method).

Population (col.) vs. Prediction (Row)	Positive (%)	Negative (%)
Positive (%)	44.6/44.6	11.6/13.8
Negative (%)	5.3/5.4	38.4/36.2

**Table 5:** Performance of the outcomes for the joint methods. Empty cells correspond to impossible combinations of outcomes.

Local (row) vs. Global (col.)	TP (%)	FP (%)	FN (%)	TN (%)
TP(%)	42.1		2.5	
FP(%)		9.7		1.9
FN(%)	2.5		2.9	
TN(%)		4.1		34.3

**Table 6:** Correct vs. wrong predictions (joint models).

Local (row) vs. Global (col.)	Correct Predictions (%)	Wrong Predictions (%)
correct predictions (%)	76.4	6.6
wrong predictions (%)	4.4	12.6

## Data Availability

Not applicable.
